# Dihydroartemisinin Exerts Anti-Tumor Activity by Inducing Mitochondrion and Endoplasmic Reticulum Apoptosis and Autophagic Cell Death in Human Glioblastoma Cells

**DOI:** 10.3389/fncel.2017.00310

**Published:** 2017-09-29

**Authors:** Chengbin Qu, Jun Ma, Xiaobai Liu, Yixue Xue, Jian Zheng, Libo Liu, Jing Liu, Zhen Li, Lei Zhang, Yunhui Liu

**Affiliations:** ^1^Department of Neurosurgery, Shengjing Hospital of China Medical University, Shenyang, China; ^2^Liaoning Clinical Medical Research Center in Nervous System Disease, Shenyang, China; ^3^Liaoning Key Laboratory of Neuro-Oncology, Shenyang, China; ^4^Department of Neurobiology, College of Basic Medicine, China Medical University, Shenyang, China; ^5^Key Laboratory of Cell Biology, Ministry of Public Health of China, China Medical University, Shenyang, China; ^6^Key Laboratory of Medical Cell Biology, Ministry of Education of China, China Medical University, Shenyang, China

**Keywords:** dihydroartemisinin, reactive oxygen species, mitochondrion, endoplasmic reticulum, apoptosis, autophagy, glioblastoma

## Abstract

Glioblastoma (GBM) is the most advanced and aggressive form of gliomas. Dihydroartemisinin (DHA) has been shown to exhibit anti-tumor activity in various cancer cells. However, the effect and molecular mechanisms underlying its anti-tumor activity in human GBM cells remain to be elucidated. Our results proved that DHA treatment significantly reduced cell viability in a dose- and time-dependent manner by CCK-8 assay. Further investigation identified that the cell viability was rescued by pretreatment either with Z-VAD-FMK, 3-methyladenine (3-MA) or in combination. Moreover, DHA induced apoptosis of GBM cells through mitochondrial membrane depolarization, release of cytochrome c and activation of caspases-9. Enhanced expression of GRP78, CHOP and eIF2α and activation of caspase 12 were additionally confirmed that endoplasmic reticulum (ER) stress pathway of apoptosis was involved in the cytotoxicity of DHA. DHA-treated GBM cells exhibited the morphological and biochemical changes typical of autophagy. Co-treatment with chloroquine (CQ) significantly induced the above effects. Furthermore, ER stress and mitochondrial dysfunction were involved in the DHA-induced autophagy. Further study revealed that accumulation of reactive oxygen species (ROS) was attributed to the DHA induction of apoptosis and autophagy. The illustration of these molecular mechanisms will present a novel insight for the treatment of human GBM.

## Introduction

Glioma is the most common and aggressive type of primary adult brain tumor. Among them, glioblastoma (GBM) is the most advanced, aggressive and frequent form of glioma, and its highly malignant and invasive nature gives rise to a median survival of only 15 months for patients with GBM undergoing conventional treatment (Kleihues et al., 1995; Griscelli et al., 2000). Thus, searching for effective therapies toward GBM remains an exciting area of investigation.

Dihydroartemisinin (DHA), a semi-synthetic derivative of artemisinin isolated from the traditional Chinese herb Artemisia annua, has been widely used as an effective anti-malarial drug (Klayman, 1985; AlKadi, 2007). In addition to this efficacy, DHA has recently been proved to possess anti-tumor activities in several human cancers, including breast, colorectal, ovarian, hepatic, pancreatic and prostate cancer, which demonstrates that DHA can induce apoptosis and/or inhibit the proliferation of cancer cells (Hou et al., 2008; Morrissey et al., 2010; Zhou et al., 2013; Feng et al., 2014; Ontikatze et al., 2014; Lucibello et al., 2015). Furthermore, it has also been confirmed that DHA exerts cytotoxicity in rat C6 glioma cells (Huang et al., 2007). However, the cytotoxicity and the underlying mechanisms of DHA in human GBM remain to be further investigated.

Apoptosis and autophagy are the two main factors involved in the anti-tumor process of chemotherapeutics. Many anticancer drugs exert the cytotoxicity in cancer cells by inducing apoptosis (Arko et al., 2010; Morrissey et al., 2010). Autophagy, known as type II programmed cell death, is a process consisting of the degradation and recycling of organelles and portions of the cytosol, which is regarded both as a cell survival and death mechanism determined by the cellular context and treatment conditions (Knizhnik et al., 2013). Recent studies strongly supported that the activation of autophagic cell death was a possible tumor suppression mechanism, which suggested cell death could occur associated with features of autophagy (Kroemer and Levine, 2008; Salazar et al., 2009; Liu and Levine, 2015). Previous studies have found that DHA induced apoptosis in human gastric cancer and prostate cancer cells (Xu G. et al., 2016; Zhang et al., 2017) and activated the autophagy program in several cancer cells (Hu et al., 2014). However, whether DHA induces apoptosis and autophagy in human GBM cells needs to be explored urgently.

Reactive oxygen species (ROS) are generally derived from the normal metabolism of oxygen (Kuo et al., 2010). At low concentrations, ROS serve as a physiological regulator of normal cell proliferation and differentiation. However, the up-regulation or decreased removal of intracellular ROS induces oxidative damage to cells and tissues (Klaunig et al., 2011). It is well known that elevated ROS can induce apoptosis—usually depending on the level of intracellular ROS and ATP (Ryter et al., 2007). Recent studies also demonstrated that ROS could induce autophagic cell death (Azad et al., 2009). Moreover, DHA has been revealed to contain an endoperoxide bridge, which has been confirmed to react with iron to form ROS (Jia et al., 2014).

Based on these studies, we primarily investigated: (i) whether DHA has cytotoxic effect on human GBM cells; (ii) what is the role of apoptosis and autophagy in the anti-tumor activity of DHA in human GBM cells; (iii) whether DHA induces ROS accumulation in human GBM cells; (iv) whether induction of apoptosis and autophagy is dependent on ROS generation in DHA treated GBM cells. The probe into the above molecular mechanisms will present a novel insight into the mechanisms of antitumor activity induced by DHA in human GBM cells.

## Materials and Methods

### Reagents and Antibodies

DMEM and fetal bovine serum (FBS) were purchased from Gibco (Carlsbad, CA, USA). DHA was purchased from ShangHai YuanYe Biotechnology Co., Ltd (Shanghai, China). The stock solution of DHA was made by dissolving 284 mg DHA in 1 mL dimethyl sulfoxide (DMSO) at a final concentration of 1 mM, which was further diluted to appropriate concentrations (0–600 μM) with cell culture medium immediately before use (the final DMSO concentration was <0.1%). Z-VAD-FMK, 3-methyladenine (3-MA) and MnTMPyP were purchased from PeproTech (St. Louis, MO, USA). Chloroquine (CQ) and mitochondria isolation kit were purchased from Sigma (St. Louis, MO, USA). Mitochondrial membrane potential (MMP) assay kit and CCK-8 was purchased from Beyotime (Jiangsu, China). Annexin V-FITC apoptosis detection kit was from BD (San Jose, CA, USA). Antibodies used were listed in Table 1.

**Table 1:** Information for antibodies.

Antibody name	Manufacturer	Catalog	Application	Dilutions
LC3	Abcam	ab63817	Western blot	1:1000
			Immunofluorescence staining	1:200
p62/SQSTM1	Abcam	ab56416	Western blot	1:1000
Beclin-1	Abcam	ab62557	Western blot	1:1000
GRP78	Cell Signaling Technology	3183	Western blot	1:1000
CHOP	Cell Signaling Technology	5554	Western blot	1:1000
eIF2α	Cell Signaling Technology	5324	Western blot	1:1000
cytochrome C	Santa Cruz Biotechnology	sc-13561	Western blot	1:1000
caspase3	Santa Cruz Biotechnology	sc-271759	Western blot	1:1000
caspase8	Santa Cruz Biotechnology	sc-5263	Western blot	1:1000
caspase9	Santa Cruz Biotechnology	sc-17784	Western blot	1:1000
caspase12	Santa Cruz Biotechnology	sc-515103	Western blot	1:1000
TOMM20	BD Biosciences	612278	Western blot	1:1000
TIMM23	BD Biosciences	611222	Western blot	1:1000
GAPDH	Santa Cruz Biotechnology	sc-365062	Western blot	1:1000
COX IV	Abcam	ab33985	Western blot	1:1000
Secondary antibodies	Santa Cruz Biotechnology	sc-2005	Western blot	1:5000
		sc-2004
Alexa Fluor 488 conjugated secondary antibody	Abcam	ab150077	Immunofluorescence staining	1:500
		ab150117

### Cell Line and Cell Cultures

Human GBM cell lines U87 and U251 cells were obtained from Shanghai Institutes for Biological Sciences and Cell Resource Center. Cells were cultured in DMEM with high glucose supplemented with 10% FBS in a humidified atmosphere of 5% CO_2_ at 37°C.

### Cell Viability Assay

Cell viability was measured by CCK-8. Cells were seeded in the 96-well culture plate at 8 × 10^3^ cells/well. After overnight incubation, cells were rinsed with PBS and incubated in complete medium with different concentrations of DHA (0.2, 2, 20, 50, 100, 200 and 600 μmol/L) for 24 h, 48 h and 72 h, respectively. Afterwards, 10 μL of CCK8 was added to each well and then plates incubated for additional 2 h. Absorbance was measured at 450 nm with a Thermo Varioskan Flash reader. For the group of pretreatment with autophagy inhibitor (3-MA, 2 mM), caspase inhibitor (Z-VAD-FMK, 100 μM), lysosome inhibitor (CQ, 15 μM) and SOD mimic (MnTMPyP, 2 mM), cells were treated with the above drugs for 24 h before DHA treatment, and other procedure was the same as above.

### Detection of Cell Apoptosis by Flow Cytometry

After treated with DHA, cells were collected and resuspended in 200 μL of binding buffer. After adding 10 μL Annexin V-FITC, cells were incubated at room temperature in the dark for 15 min. After that, 300 μL of binding buffer and 5 μL of PI were added, and the samples were immediately measured using a flow cytometer (FACScan, BD Biosciences, San Jose, CA, USA). The results were analyzed using CELLQuest 3.0 software.

### Mitochondrial Membrane Potential (MMP) Assay

MMP was measured using the MMP kit, according to the method previously described (Ma et al., 2015). Cells were harvested by centrifugation and stained with 10 μg/mL JC-1 for 20 min in the dark at 37°C. CytoFluor plate reader (excitation wavelength of 485 nm, slit width of 20 nm) was used to monitor the fluorescence intensities for the monomer and the aggregated JC-1 molecules (emission wavelengths of 520 nm, with slit width of 25 nm, and 580 nm, with slit width of 30 nm, respectively). The results were analyzed by cell quest software.

### Transmission Electron Microscopy (TEM) Assay

To morphologically demonstrate the induction of autophagy in DHA treated GBM cells, the ultrastructural analysis was performed by transmission electron microscopy (TEM) assay. Cells were fixed with 2.5% glutaraldehyde overnight at 4°C and subsequently fixed with 1% OsO_4_-0.15 M Na cacodylate/HCl. Then, the samples were dehydrated in graded ethanol and polymerized, and subjected to the electron microscopy analysis.

### Immunofluorescence Staining

Treated cells were stained with LysoTracker Red (50 nM), fixed in 4% paraformaldehyde for 30 min, permeabilized with 0.1% Triton X-100 for 10 min at room temperature, and blocked with PBS containing 5% bovine serum albumin for 2 h. Following incubation with antibodies against LC3 overnight at 4°C, cells were treated with Alexa Fluor 488 conjugated secondary antibody for 2 h at room temperature. The cells were then counterstained with DAPI and the punctuate pattern was visualized under the confocal microscopy.

### Mitochondria Isolation

Mitochondrial extracts were isolated using mitochondria isolation kit, according to the manufacturer’s instructions. In brief, treated cells were washed with PBS and resuspended with ice-cold extraction buffer supplemented with cell lysis detergent and PMSF. Afterwards, cells were added extraction buffer and centrifuged twice at 2000 *g* for 10 min at 4°C. The supernatants were collected in a new tube, and centrifuged at 11,000 *g* for 10 min at 4°C. Supernatants were discarded, and the pellets containing the mitochondrial fraction washed with extraction buffer and centrifuged. The mitochondrial fraction were stored at −80°C.

### Western Blot Assay

Treated cells were washed with cold PBS and lysed in radio immunoprecipitation assay (RIPA) buffer supplemented with a proteinase inhibitor for extracting total protein. Protein concentration was determined by the bicinchoninic acid (BCA) protein assay. After denatured, proteins were separated in SDS polyacrylamide gel electrophoresis and transferred onto PVDF membranes. Nonspecific binding was blocked with 5% milk in TBST buffer for 2 h, followed by incubation with primary antibodies at 4°C overnight and secondary antibodies at room temperature for 2 h. Blots were visualized using ECL detection reagents. Integrated light density values (IDVs) were calculated by Fluor Chen 2.0 software.

### ROS Measurement

ROS levels were detected based on the oxidation of DCFH-DA by peroxide to produce the fluorescent product 2′,7′-dichlorofluorescein (DCF), as previously described (Chang et al., 2010). In brief, treated cells were washed and incubated with DCFH-DA at a final concentration of 10 μM for 30 min. After washing, cells were applied to flow cytometry using 488 nm excitation and 530 nm emission wavelengths. The mean DCFH-DA fluorescence intensity was determined using FlowJo 7.6 software.

### Statistical Analysis

Data are expressed as mean ± standard deviation (SD). Statistical Package for Social Sciences software (SPSS 19.0) was used for statistical analyses. Statistical significance was calculated using the Student’s *t*-test or one-way analysis of variance (ANOVA). Differences were considered significant if *P* < 0.05.

## Results

### DHA Possessed Cytotoxic Effects on Human GBM Cells

**Figure 1:**
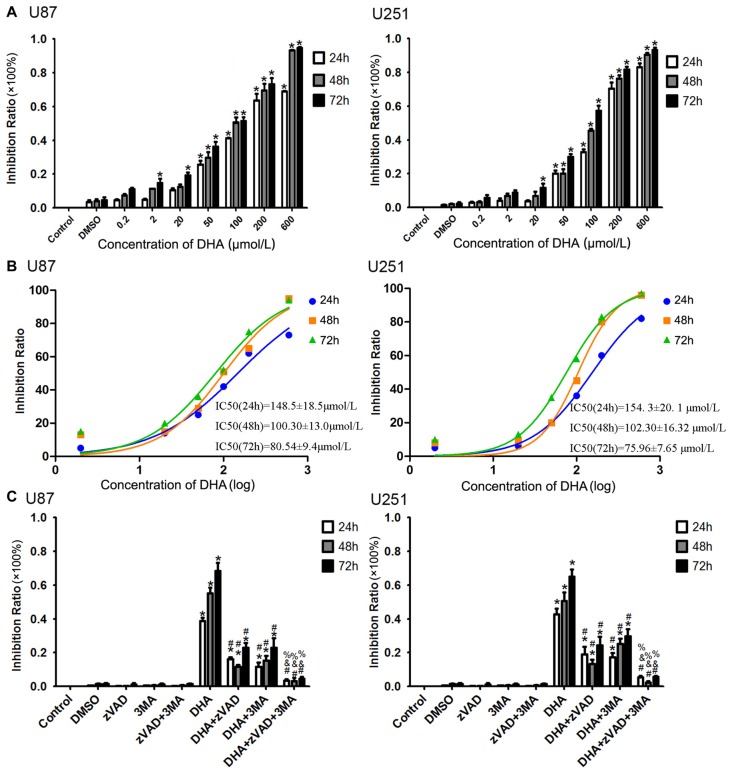
Dihydroartemisinin (DHA) possessed cytotoxic effects on human glioblastoma (GBM) cells. **(A)** DHA reduced the cell viability in a dose and time-dependent manner both in U87 and U251 cells. The cell viability were significantly inhibited in U87 and U251 cells treated with 50, 100, 200 and 600 μM DHA in 24 h, 48 h and 72 h. The inhibition rate was calculated using the following formula: 1−Experimental group/Control group × 100%. **(B)** The non-linear regression curve analysis of the concentration-effect responses relative to the DHA treatment at 24 h, 48 h and 72 h were calculated, and the F-test was perform for data analysis among the IC50 of the 24 h, 48 h and 72 h curves. **(C)** The cell viability pretreated with either Z-VAD-FMK, 3-methyladenine (3-MA) or in combination was markedly ameliorated. The inhibition rate by pretreatment with both Z-VAD-FMK and 3-MA was no different from the control group. Data are expressed as means ± standard deviation (SD; *n* = 5, each). *P < 0.05 vs. control group, ^#^*P* < 0.05 vs. DHA group, ^&^*P* < 0.05 vs. DHA+Z-VAD group, ^%^*P* < 0.05 vs. DHA+3-MA group.

After human U87 and U251 GBM cells treated as mentioned above, the cells were first subjected to CCK-8 assay. As shown in Figure 1A, DHA reduced the cell viability in a dose and time-dependent manner. The cell viability of U87 and U251 cells were decreased with the DHA concentration increasing, and decreased with the DHA-treated time increasing. There was no significant in U87 and U251 cells treated with 0.2 μM DHA at 24 h, 48 h and 72 h. In addition, there was no significant in U87 cells treated with 2 μM DHA at 24 h and 48 h, whereas cell viability was significantly inhibited at 72 h. However, there was no significant in U251 cells treated with 2 μM DHA at 24 h, 48 h and 72 h. In cells treated with 20 μM DHA at 24 h, 48 h and 72 h, the U87 and U251 cell viability was significantly inhibited in 72 h. Furthermore, the cell viability were significantly inhibited in U87 and U251 cells treated with 50, 100, 200 and 600 μM DHA in 24 h, 48 h and 72 h. The IC50 values of DHA in U87 cells at 24 h, 48 h and 72 h was 148.5 ± 18.5 μmol/L, 100.30 ± 13.0 μmol/L and 80.54 ± 9.4 μmol/L, respectively. Meanwhile, the IC50 values of DHA in U251 cells at 24 h, 48 h and 72 h was 154. 3 ± 20. 1 μmol/L, 102.30 ± 16.32 μmol/L and 75.96 ± 7.65 μmol/L, respectively. There were difference among the IC50 values of DHA in U87 or U251 cells at 24 h, 48 h and 72 h (Figure 1B). Therefore, 100 μM of DHA was selected as the optimal administration concentration in the subsequent experiments.

To investigate whether apoptosis and autophagy were involved in the cytotoxicity of DHA, cells were pretreated with caspase inhibitor (Z-VAD-FMK), autophagy inhibitor (3-MA) or in combination. There was no significant difference in Z-VAD-FMK, 3-MA or in combination groups compared with the control group. The cell viability was reduced in cells treated with 100 μM of DHA at 24 h, 48 h and 72 h. Additionally, the cell viability of DHA+Z-VAD, DHA+3-MA and DHA+Z-VAD+3-MA groups were up-regulated compared with DHA group, suggesting that Z-VAD and 3-MA blocked the inhibitory effect of DHA on the cell viability. Further, the cell viability of DHA+Z-VAD+3-MA group was up-regulated compared with DHA+Z-VAD or DHA+3-MA groups. These results demonstrated that the cell viability of U87 and U251 cells pretreated with either *Z-VAD-FMK* or 3-MA was markedly ameliorated (Figure 1C). The above results suggested that the cytotoxic effects of DHA on human GBM cells was associated with cell apoptosis and autophagy.

### DHA Induced Apoptosis through Mitochondria and Endoplasmic Reticulum (ER) Stress Pathways of Apoptosis in Human GBM Cells

**Figure 2:**
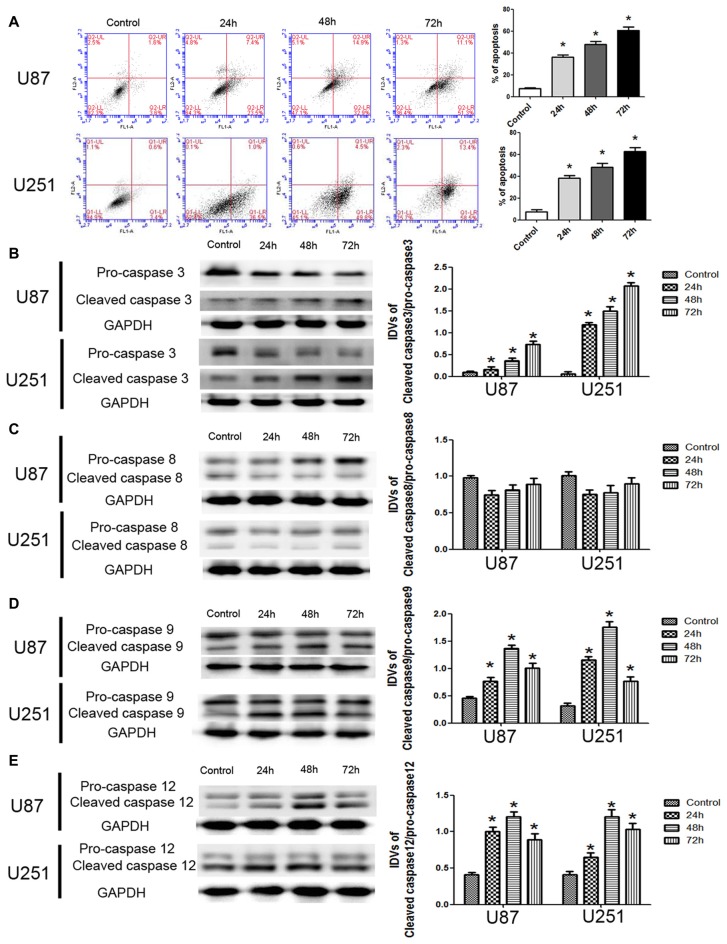
DHA induced apoptosis by regulating caspase family proteins in human GBM cells. **(A)** Flow cytometric analysis was used to detect apoptotic rate in the GBM cells treated with DHA. The significantly enhanced apoptotic cells by DHA treatment were detected compared with the control group. The early and late apoptosis was calculated as the % of apoptosis. **(B)** Western blot analysis of the expression levels of active cleaved caspase-3 in GBM cells after treatment with DHA. The expression of active caspase-3 was increased in DHA treated GBM cells. **(C)** Western blot analysis of the expression levels of active cleaved caspase-8 in GBM cells after treatment with DHA. The expression of active caspase-8 was no change. Western blot analysis of the expression levels of active cleaved caspase-9 **(D)** and caspase-12 **(E)** in GBM cells after treatment with DHA. The expression of active cleaved caspase-9 and caspase-12 were increased in DHA treated GBM cells. Data are expressed as means ± SD (*n* = 5, each). *P < 0.05 vs. control group.

To further support, apoptosis was involved in the cytotoxic effects of DHA in GBM cells, we performed the flow cytometric analysis. As shown in Figure 2A, the apoptosis was induced in U87 and U251 cells treated with DHA at 24 h, 48 h and 72 h. Consistent with this result, the expression of active cleaved caspase-3 was increased in DHA treated cells at 24 h, 48 h and 72 h (Figure 2B). As there are three classical apoptotic pathways, we further investigated which apoptotic pathway was involved in the anti-tumor activity of DHA in GBM cells. We found that there was no change of the active caspase-8 expression in cells treated with DHA at 24 h, 48 h and 72 h, suggesting that the extrinsic apoptotic pathway was not involved in the anti-tumor activity of DHA (Figure 2C). However, the active cleaved caspase-9 and caspase-12 expression were increased in DHA treated cells, indicating that DHA promoted mitochondrion and endoplasmic reticulum (ER) apoptosis in human GBM cells (Figures 2D,E).

**Figure 3:**
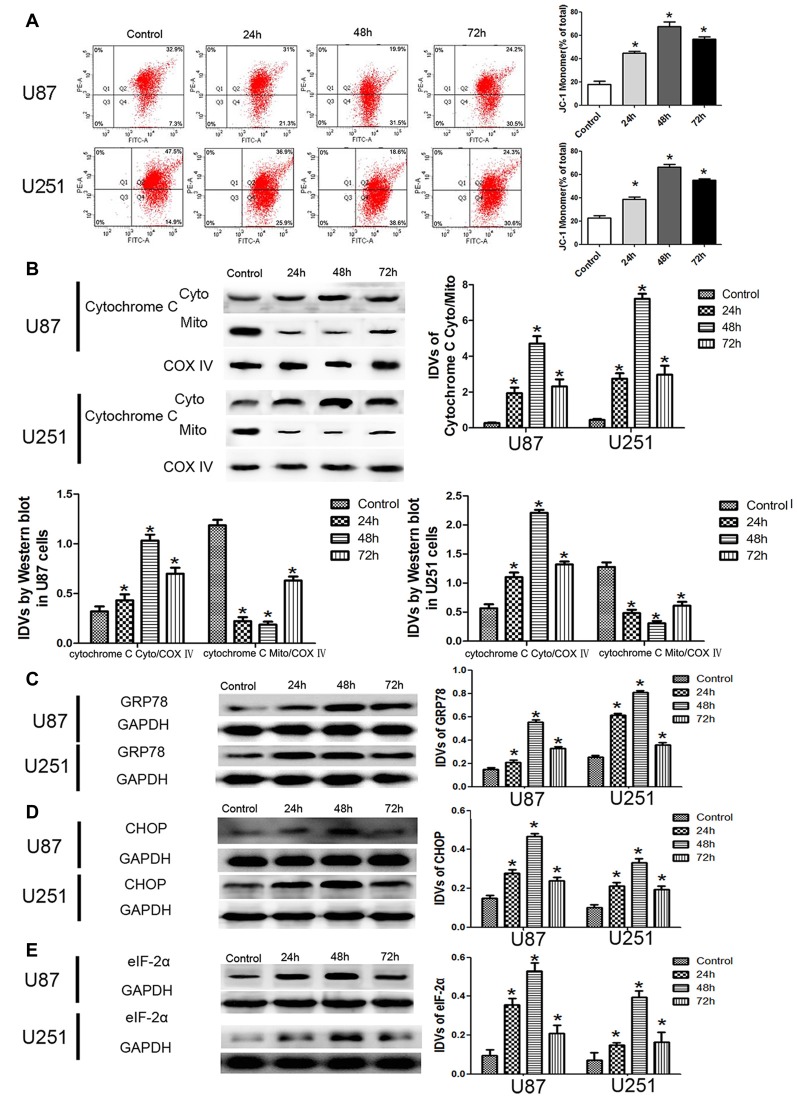
DHA induced apoptosis by regulating cytosolic cytochrome C and UPR-related proteins (GRP78, eIF2α and CHOP) in human GBM cells. **(A)** Effects of DHA on the mitochondrial membrane potential (MMP) of GBM cells by JC-1 staining for flow cytometry. DHA treatment significantly reduced the MMP of GBM cells. **(B)** The expression of cytosolic and mitochondrial cytochrome C in cells treated with DHA was checked by Western blot assay. The expression of cytosolic cytochrome C was increased. The integrated light density values (IDVs) of cytochrome C are shown using COX IV as an endogenous control. The IDVs was calculated as the ratio of cytochrome C Cyto/Mito, cytochrome C Cyto/COX IV and cytochrome C Mito/COX IV. **(C–E)** Western blot analysis was performed to detect the expression levels of GRP78, CHOP and eIF2α in GBM cells after treatment with DHA. The expression of GRP78, CHOP and eIF2α was enhanced in DHA treated GBM cells. Data are expressed as means ± SD (*n* = 5, each). *P < 0.05 vs. control group.

JC-1 staining was used to further detect the effect of DHA on the MMP of GBM cells. As shown in Figure 3A, JC-1 monomer was increased in DHA treated cells at 24 h, 48 h and 72 h, suggesting that DHA reduced the MMP of GBM cells. Moreover, the cytosolic cytochrome C was increased in DHA treated cells at 24 h, 48 h and 72 h (Figure 3B). These results indicated that the mitochondrial pathway of apoptosis was induced in DHA treatment. Furthermore, the up-regulated expression of GRP78, CHOP and eIF2α were also detected in DHA treated cells (Figures 3C–E), suggesting that ER stress apoptosis was induced in the anti-tumor activity of DHA. The above results demonstrated that DHA activated the mitochondria and ER stress pathway of apoptosis in human GBM cells.

### Induction of Complete Autophagy in DHA Treated Human GBM Cells

**Figure 4:**
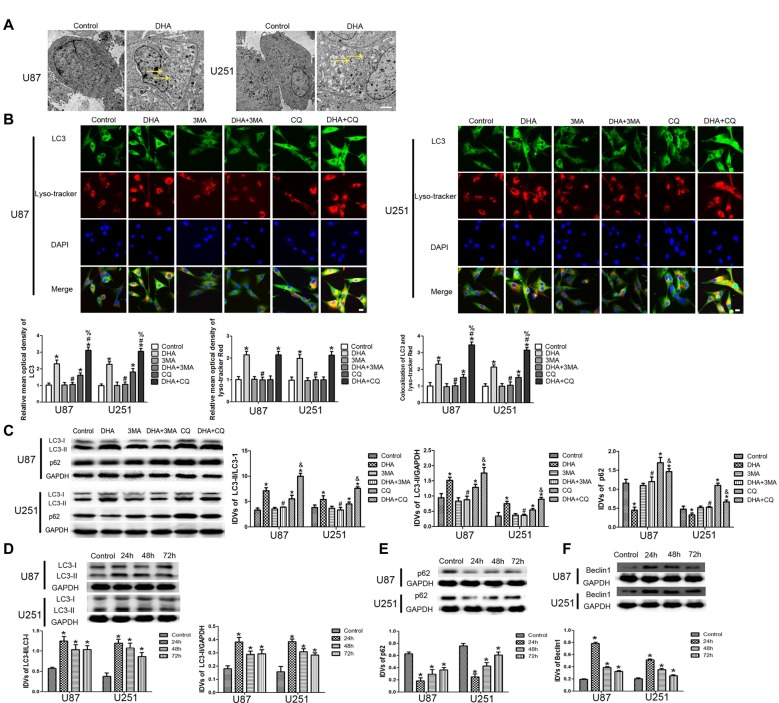
Induction of complete autophagy in DHA treated human GBM cells. **(A)** Electron microscopy showed autophagic vacuoles (AVs) in DHA treated GBM cells. **(B)** The colocalization of LC3 and LysoTracker Red in GBM cells. Relative mean optical density of LC3 and LysoTracker Red, and the co-localization of LC3 and LysoTracker Red were quantified. Pictures are respective magnification (*n* = 4, each). Scale bar = 20 μm. **(C)** The expression levels of LC3-II/LC3-I, LC3-II/GAPDH, p62/SQSTM1 in the GBM cells after treatment with DHA, 3-MA and chloroquine (CQ). Data are expressed as means ± SD (*n* = 5, each), *P < 0.05 vs. control group, ^#^*P* < 0.05 vs. DHA group, ^%^*P* < 0.05 or ^&^*P* < 0.05 vs. CQ group. Western blot analysis was performed to detect the expression levels of **(D)** LC3-II/LC3-I and LC3-II/GAPDH, **(E)** p62/SQSTM1 and **(F)** Beclin-1 in GBM cells after treatment with DHA. Data are expressed as means ± SD (*n* = 5, each), *P < 0.05 vs. control group.

To confirm whether DHA induces autophagy in GBM cells, we first performed TEM assay. As shown in Figure 4A, the autophagic vacuoles (AVs) were positive in U87 and U251 cells treated with DHA. Immunofluorescence staining for LC3 revealed the presence of LC3-positive cytoplasmic inclusions. Since both autophagosome formation and impaired autophagosomes degradation ascribes to increased AVs, the effect of inhibiting lysosomal turnover of autophagosome contents by CQ were also examined. As shown in Figure 4B, higher magnification of punctate aggregates were found in DHA, CQ and DHA+CQ treated GBM cells compared with the control group. Co-treatment with 3-MA reduced the punctate distribution and density of LC3. In addition, CQ markedly increased the numbers of AVs elicited by DHA exposure, indicating that autophagy induced by DHA was complete. These results suggested that DHA may increase LC3 levels by induction of LC3-II formation at an early step, and not by blocking degradation of autophagosomes at a later step, indicating that autophagy induced by DHA was complete.

The expression of LC3-II and p62/SQSTM1 were detected after cells treated with DHA, 3-MA and CQ. Results showed that the expression of LC3-II was increased in DHA, CQ and DHA+CQ groups compared with the control group, and was reduced in DHA+3-MA group compared with DHA group. Moreover, the LC3-II expression was increased in DHA+CQ group compared with the CQ group. The expression of p62/SQSTM1 was reduced in DHA group and increased in CQ and DHA+CQ groups compared with the control group, and was increased in DHA+3-MA group compared with DHA group. Moreover, the p62/SQSTM1 expression was reduced in DHA+CQ group compared with the CQ group (Figure 4C). Further, the expression of autophagy-related genes, two MAP1-LC3 forms (LC3-I and LC3-II), p62/SQSTM1 and Beclin-1 were detected by Western blot, and results showed that increased conversion of LC3-I to LC3-II and increased Beclin-1 expression were observed in DHA treated GBM cells, whereas the reduced expression of p62/SQSTM1 was found in DHA treated GBM cells (Figures 4D–F). These results demonstrated that DHA induced complete autophagy in human GBM cells.

### ER Stress and Mitochondrial Dysfunction Were Involved in the DHA-Induced Autophagy

**Figure 5:**
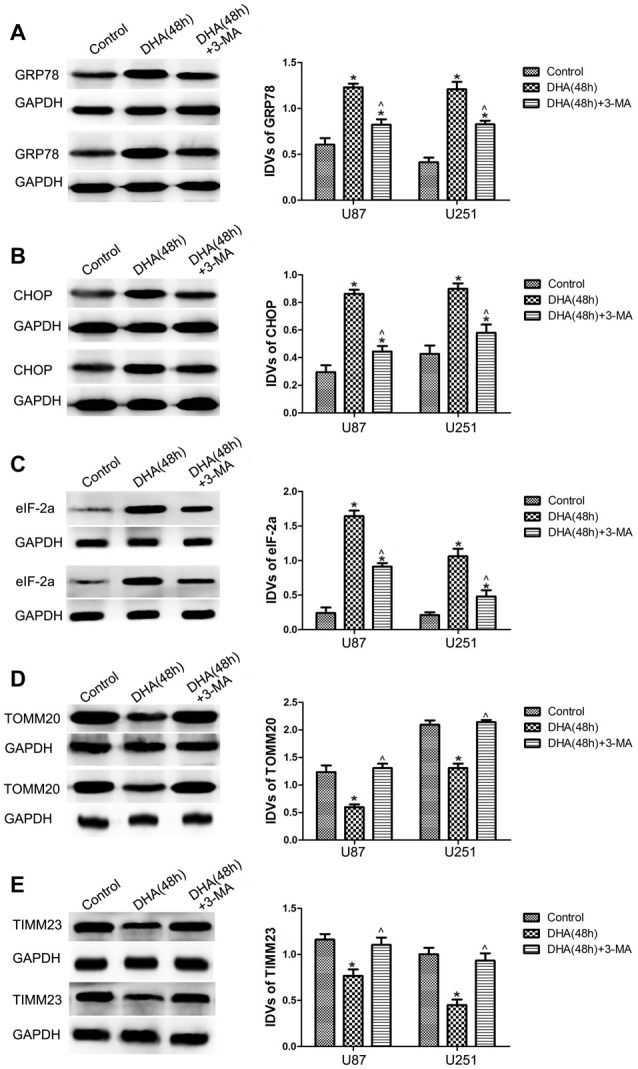
Endoplasmic reticulum (ER) stress and mitochondrial dysfunction were involved in the DHA-induced autophagy. Western blot analysis was performed to detect the expression of **(A)** GRP78, **(B)** CHOP and **(C)** eIF2α in GBM cells after treatment with DHA, 3-MA or in combination. Western blot analysis of the expression of **(D)** TOMM20 and **(E)** TIMM23 in GBM cells after treatment with DHA, 3-MA or in combination. Data are expressed as means ± SD (*n* = 5, each), *P < 0.05 vs. control group; ^ᶺ^*P* < 0.05 vs. DHA group.

To clarify whether ER stress is involved in the autophagy induction of DHA, the expression of UPR-related proteins (GRP78, CHOP and eIF-2α) were measured in cells pretreated with 3-MA. Our results showed that DHA up-regulated the expression of GRP78, CHOP and eIF2α at 48 h in GBM cells, and cells pretreated with 3-MA partly reversed the above effect, suggesting that ER stress was involved in the DHA-induced autophagy (Figures 5A–C).

Mitophagy is a process through which dysfunctional mitochondria are selectively removed by autophagy (Kim et al., 2007). To clarify whether mitophagy is involved in the cells treated with DHA, the outer and inner mitochondrial membrane proteins (TOMM20 and TIMM23) were detected. In Figures 5D,E, DHA reduced the expression of TOMM20 and TIMM23, and co-treatment with 3-MA rescued the above inhibitory effect. These results suggested that mitochondrial dysfunction was involved in the DHA-induced autophagy.

### DHA Cytotoxicity Was Dependent on ROS Generation in Human GBM Cells

**Figure 6:**
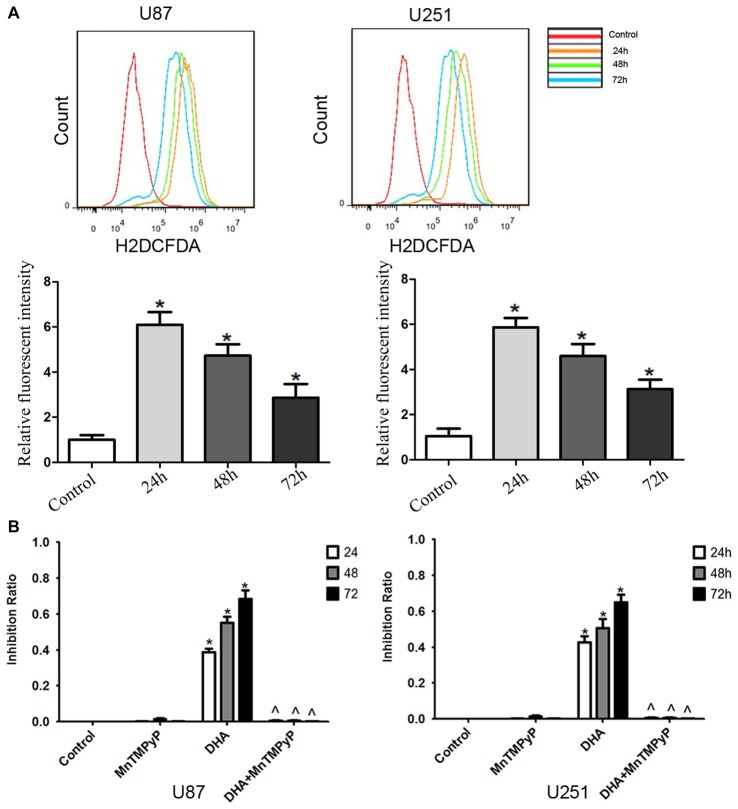
The cytotoxicity of DHA was dependent on reactive oxygen species (ROS) generation in human GBM cells. **(A)** Flow cytometry analysis revealed that DHA treatment resulted in an increased ROS levels in GBM cells. The *x*-axis represents the fluorescence intensity and the *y-axis* represents the number of cells (upper panel). The mean DCFH-DA fluorescence intensity was determined (lower panel). **(B)** Pretreated GBM cells with MnTMPyP significantly attenuated the inhibition effect on the cell viability of human GBM cells by DHA. Data are expressed as means ± SD (*n* = 5, each), *P < 0.05 vs. control group, ^ᶺ^*P* < 0.05 vs. DHA group.

Flow cytometry analysis revealed that DHA treatment resulted in an increased ROS levels in GBM cells compared with the control group (Figure 6A). Moreover, GBM cells pretreated with MnTMPyP, a SOD mimic, attenuated the inhibitory effect on the cell viability of human GBM cells by DHA (Figure 6B). These results indicated that the cytotoxicity of DHA in human GBM cells was dependent on ROS generation.

### DHA Induced Apoptosis Was Mediated by ROS Generation in Human GBM Cells

**Figure 7:**
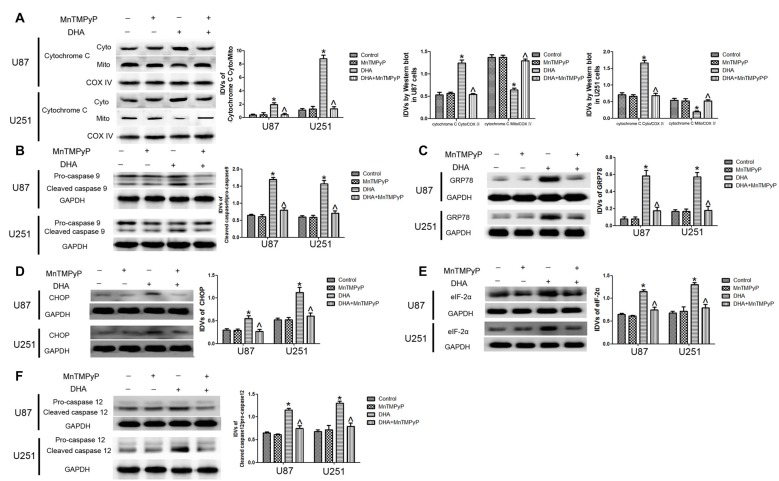
ROS generation mediated the induction of apoptosis by DHA in human GBM cells. **(A)** Western blot analysis was performed to detect the expression of cytosolic and mitochondrial cytochrome C in GBM cells treated with MnTMPyP, DHA or in combination. Cells pretreated with MnTMPyP significantly reduced the expression of cytosolic cytochrome C compared with DHA treatment alone. The IDVs of cytochrome C are shown using COX IV as an endogenous control. The IDVs was calculated as the ratio of cytochrome C Cyto/Mito, cytochrome C Cyto/COX IV and cytochrome C Mito/COX IV. **(B)** Western blot analysis was performed to detect the expression of active cleaved caspase-9 in GBM cells treated with MnTMPyP, DHA or in combination. The expression of active caspase-9 was reduced in pretreatment with MnTMPyP group compared with DHA treatment alone. **(C–F)** Western blot analysis was performed to detect the expression of GRP78, CHOP, eIF2α and active cleaved caspase-12 in GBM cells treated with MnTMPyP, DHA or in combination. Pretreatment with MnTMPyP markedly reduced the expression of GRP78, CHOP, eIF2α and active cleaved caspase-12 compared with DHA treatment alone. Data are expressed as means ± SD (*n* = 5, each), *P < 0.05 vs. control group, ^ᶺ^*P* < 0.05 vs. DHA group.

Further, we checked whether DHA-induced apoptosis is mediated by the ROS generation. As shown in Figures 7A,B, the expression of cytosolic cytochrome C and active cleaved caspase-9 were increased in GBM cells treated with DHA, and GBM cells pretreated with MnTMPyP significantly reduced these expression compared with DHA treatment alone, suggesting that DHA induced the mitochondrial pathway of apoptosis was mediated by ROS generation. Besides, the expression of GRP78, CHOP, eIF2α and active cleaved caspase-12 were up-regulated in GBM cells treated with DHA, and pretreatment with MnTMPyP markedly reduced these expression, confirming the critical role of ROS generation in the induction of ER stress pathway of apoptosis (Figures 7C–F).

### DHA Induced Autophagy Was Mediated by ROS Generation in Human GBM Cells

**Figure 8:**
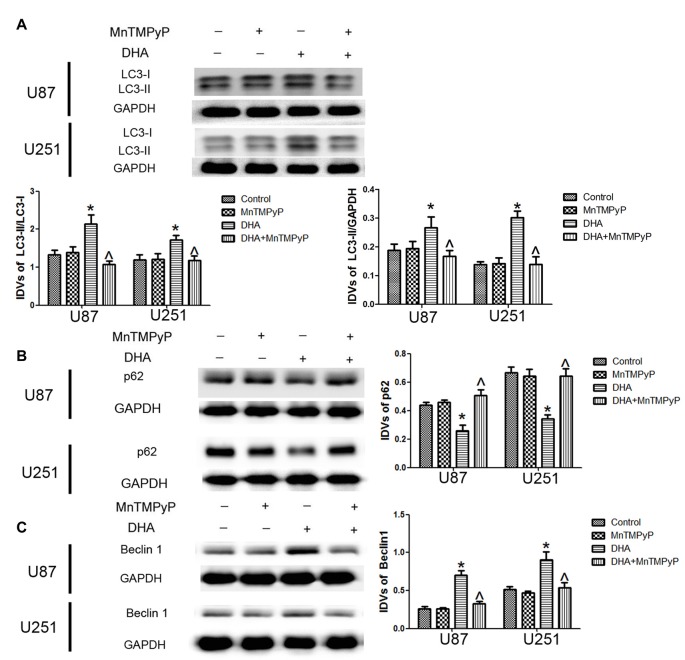
ROS generation mediated the induction of autophagy by DHA in human GBM cells. Western blot analysis was performed to detect the expression of **(A)** LC3-II/LC3-I and LC3-II/GAPDH, **(B)** p62/SQSTM1 and **(C)** Beclin-1 in GBM cells treated with MnTMPyP, DHA or in combination. The conversion of LC3-I to LC3-II and the expression of Beclin-1 after cells pretreated with MnTMPyP was obviously reduced compared with DHA treatment alone. The expression of p62/SQSTM1 was enhanced after cells pretreated with MnTMPyP compared with DHA treatment alone. Data are expressed as means ± SD (*n* = 5, each), *P < 0.05 vs. control group, ^ᶺ^*P* < 0.05 vs. DHA group.

We further detected whether DHA-induced autophagy is mediated by the ROS generation, and results showed that the conversion of LC3-I to LC3-II and Beclin-1 expression was increased, whereas p62/SQSTM1 expression was decreased in GBM cells treated with DHA. Moreover, the conversion of LC3-I to LC3-II after cells pretreated with MnTMPyP was obviously reduced compared with DHA treatment alone. Markedly enhanced p62/SQSTM1 expression and decreased Beclin-1 expression in MnTMPyP pretreatment cells were detected compared with DHA treatment alone (Figures 8A–C), suggesting ROS generation was contributed to the induction of autophagy by DHA in human GBM cells.

## Discussion

In the present study, we demonstrated that DHA exerted anti-tumor activity by reducing cell viability of GBM cells in a dose- and time-dependent manner, which was associated with cell apoptosis and autophagy. Moreover, DHA activated mitochondrion and ER stress apoptotic pathways and induced complete autophagy in human GBM cells, and these process was mediated by ROS generation.

The lack of effective long-term treatments for GBM highlights the need to identify new and potent anti-cancer drugs. Natural phytochemicals are drawing increasing interest as novel drugs, with cytotoxicity effects and little side effect demonstrated in certain cancers. DHA, a front-line anti-malarial herbal compound, has been proved to possess promising anti-cancer activity with low toxicity (Huang et al., 2007). DHA has recently been confirmed to exert the anti-tumor effect in several human tumors. Previous study found that 10 μmol/L DHA significantly inhibited the proliferation of human cholangio carcinoma QBC939 cells with prolonged duration of DHA treatment (Hu et al., 2015). In T-cell lyphoma Jurkat cell line, 10 μM DHA significantly decreased the cell viability. Recent study also found that DHA inhibited the proliferation of glioma cells *in vitro* (Zhang et al., 2015). Consistent with previous studies, our results showed that DHA could effectively exert the anti-tumor activity in human GBM cells, suggesting DHA would be a potent agent for the treatment of human GBM. Further, sole and combined pretreatment with caspase inhibitor (Z-VAD-FMK), autophagy inhibitor (3-MA) were used in our present study to clarify the molecular mechanisms. Our results showed that Z-VAD or 3-MA partly blocked the inhibitory effect of DHA on the cell viability, and Z-VAD combined with 3-MA completely rescued this effect, suggesting that the cytotoxic effects of DHA on human GBM cells was associated with cell apoptosis and autophagy. Consistent with our results, recent studies found that DHA induced apoptosis in human gastric cancer and colorectal cancer cells (Lu et al., 2015; Zhang et al., 2017), and effectively inhibited cell growth and induced apoptosis in human BT325 glioma cells and rat C6 glioma cells (Du et al., 2015). Furthermore, DHA possessed cytotoxic effects by inducing autophagy in human leukemia K562 cells and pancreatic cancer cells (Wang et al., 2012; Jia et al., 2014).

Induction of apoptosis constitutes an important mechanism for eradication of tumor cells by anti-cancer agents. There are three well-studied pathways that result in apoptosis. The intrinsic pathway is associated with mitochondrial outer membrane permeabilization, cytochrome c release and the activation of procaspase-9. In contrast, the extrinsic pathway proceeds independently of alteration in mitochondrial function, but is instead characterized by the ligation of cell surface death receptors via specific death ligands to generate catalytically active caspase-8. Besides, apoptosis can also be induced by ER stress through activation of caspase-12 (Beere, 2005; Mao et al., 2008). Though studies have indicated that induction of apoptosis of cancer cells by DHA might be an important mechanism responsible for the impact on tumor cell viability, there are still controversies and conundrums associated with the apoptotic pathway involved in the cytotoxicity of DHA. Several studies showed that DHA induced apoptosis depended on caspase-8 activation (Lu et al., 2010; Ji et al., 2011), whereas other studies confirmed that DHA induced apoptosis had no relation with extrinsic apoptotic pathway (Handrick et al., 2010). DHA induced apoptosis preferentially via intrinsic pathway in hepatocarcinoma cells (Qin et al., 2015), and induced apoptosis through extrinsic and intrinsic apoptotic pathways in human osteosarcoma (Ji et al., 2011). In addition, DHA-induced apoptosis is dependent on the extrinsic and intrinsic apoptotic pathways as well as ER stress-mediated apoptotic pathway in human lung adenocarcinoma cells (Lu et al., 2010; Chen et al., 2012). In our present study, DHA-induced apoptosis without caspase-8 activation in human GBM cells, and was partially associated with mitochondrial membrane depolarization, release of cytochrome c and activation of caspases-9. We also identified that ER stress-mediated apoptotic pathway was simultaneously involved in the cytotoxicity of DHA in human GBM cells. However, the molecular mechanisms in the different DHA-induced apoptotic pathways in different cancers needs to be further investigated.

Autophagy is an evolutionarily conserved cellular degradation pathway for various long-lived proteins and organelles. In response to stress conditions, parts of the cytoplasm or organelles are sequestered into double-membrane structures termed autophagosomes or AVs which are subsequently fused with lysosomes, resulting in degradation and recycling to allow cell survival (Yorimitsu and Klionsky, 2005). However, high levels of autophagy can function as a cell death effector mechanism. Autophagic cell death, which is different from the above apoptosis, has independent morphological and biochemical features (Kroemer et al., 2009). It has been confirmed that DHA induced autophagy in several human cancer cells (Hu et al., 2014). However, the role of autophagy in the anti-tumor activity of DHA is still controversial. Though some studies identified that autophagy induced by DHA was protective mechanism in cancers (Ganguli et al., 2014), others also confirmed that the induction of autophagy by DHA enhanced the anti-tumor activity (Feng et al., 2014). DHA triggered autophagy by up-regulating the Beclin 1 expression to induce cell death in pancreatic cancer cells (Jia et al., 2014), and stimulated autophagy through repression of NF-κB activity to induce cell death in human multiple myeloma cancer cells (Hu et al., 2014). In our present study, for the first time, we identified that DHA induced autophgy in human GBM cells, accompanied by the up-regulation of LC3II/I and Beclin-1 expression as well as the down-regulation of p62/SQSTM1 expression. Moreover, we verified that autophagy induced by DHA in GBM cells was complete by using lysosome inhibitor CQ. Our results demonstrated that autophagic cell death was partially contributed to the cytotoxicity of DHA in human GBM cells. Comprehensive analysis of others reports and our findings suggested that DHA-induced autophagy might be critical in inducing tumor cells death. Autophagy can also be initiated in response to ER stress, and cells respond to ER stress by activating a highly conserved unfolded protein response (UPR; Tsai and Weissman, 2010). Our results showed that ER stress was involved in the DHA-induced autophagy. Moreover, mitochondria has been described as a source of autophagosome biogenesis, and plays a key role in the cross-talk between autophagy and apoptosis regulation (Hailey et al., 2010; Strappazzon et al., 2011). Mitophagy is a process through which dysfunctional mitochondria are selectively removed by autophagy (Kim et al., 2007). Our results showed that mitophagy was also involved in the DHA-induced autophagy. Combined with the above results, these results suggested that ER stress and mitochondrial dysfunction were both involved in the apoptosis and autophagy induced by DHA in GBM cells.

**Figure 9:**
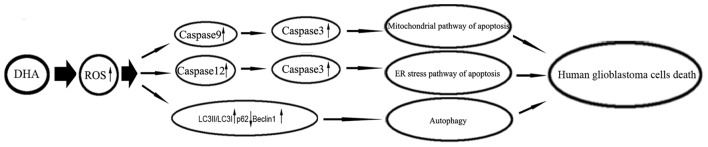
The schematic cartoon of the mechanism underlying anti-tumor activity of DHA in human GBM cell lines.

Under metabolic processes, cells can generate a partially reduced form of oxygen referred to as ROS (Ryter et al., 2007). Elevated ROS elicited a variety of oxidative stress responses ranging from cell proliferation to growth arrest, and to cell death (Martindale and Holbrook, 2002). DHA has been shown to contain an endoperoxide bridge, which was confirmed to react with iron and form ROS (Jia et al., 2014). The role of ROS as important mediators for the apoptotic signaling pathway is well supported, and revealed that the excessive elevation of ROS in tumor cells can stimulate downstream proteins and induce tumor cells death (Suzuki et al., 1997). DHA up-regulated ROS to increase ferrous ion in cells, and contributed to the apoptosis of human lung carcinoma cells (Xu C. C. et al., 2016). DHA increasing ROS resulted in the accumulation of intracellular Ca^2+^, and promoted the mitochondria and ER stress pathway of apoptosis in human colorectal cancer cells (Lu et al., 2015). Furthermore, previous study found that DHA induced autophagy and inhibited the growth of iron-loaded human myeloid leukemia cells via ROS toxicity. Consistent with these studies, our results showed that ROS generation inhibition by MnTMPyP markedly reduced the expression of cytosolic cytochrome C and active cleaved caspase-9, the expression of GRP78, CHOP, eIF2a and active cleaved caspase-12, as well as the expression of LC3-II and Beclin1, which suggested that DHA-induced apoptosis and autophagy was dependent on ROS generation in human GBM cells. Based on these notions, however, there has been no report so far to identify whether ROS act as key regulators in DHA-triggered autophagy or whether ROS generation is simply a concomitant phenomenon with autophagy in DHA-treated GBM cells. The mechanism underlying anti-tumor activity of DHA in human GBM cell lines is schematically presented in Figure 9.

In conclusion, our results demonstrated that DHA exerted the anti-tumor role through inducing ROS-mediated mitochondrion and ER stress apoptotic pathways and autophagic cell death in human GBM cells. This study will provide further support that DHA has great potential to be developed as a novel therapeutic agent for the treatment of human GBM.

## Author Contributions

YL, YX, CQ and JM: conceived and designed the experiments; CQ, JM and XL: performed the experiments; JZ, LL, JL and ZL: analyzed the data; ZL and LZ: contributed reagents/materials/analysis tools; CQ, JM, XL, YX and YL: wrote the manuscript.

## Conflict of Interest Statement

The authors declare that the research was conducted in the absence of any commercial or financial relationships that could be construed as a potential conflict of interest.
